# Lifestyle factors and high body mass index are associated with different multimorbidity clusters in the Brazilian population

**DOI:** 10.1371/journal.pone.0207649

**Published:** 2018-11-20

**Authors:** Januse Nogueira de Carvalho, Marianna de Camargo Cancela, Dyego Leandro Bezerra de Souza

**Affiliations:** 1 Graduate Program in Collective Health, Federal University of Rio Grande do Norte, Natal, Rio Grande do Norte, Brazil; 2 Division of Population Research, Brazilian National Cancer Institute, Rio de Janeiro, Brazil; Osakidetza Basque Health Service, SPAIN

## Abstract

Multimorbidity patterns of a population can be influenced by socioeconomic and lifestyle-related factors. Some of these factors are preventable when healthy habits are promoted to the population. This study analyzed the main grouping patterns of chronic diseases and the relationship with socioeconomic and lifestyle-related factors of the Brazilian population (over the age of 18), based on a population-based survey (2013 National Health Survey). A total of 60,202 participants were included. Cluster analysis was carried out to identify the combinations of chronic diseases. Bivariate and multivariate analyses were carried out to verify the relationship between disease clusters and independent variables, utilizing Poisson's regression with robust variance, considering a 95% confidence interval. Cluster analysis revealed four disease clusters:cardiometabolic diseases/cancer, mental/occupational diseases, musculoskeletal diseases and respiratory diseases, all significantly associated with the female gender, current/past smoking habits and overweight/obesity in multivariate analyses. These aspects must be considered when planning health services and developing strategies and guidelines for the prevention and treatment of multiple chronic conditions.

## Introduction

Noncommunicable diseases (NCDs) are characterized by a long duration, non-transmissibility between individuals and general slow evolution [1]. NCDs are responsible for two thirds of the 57 million deaths in the world, per year, with 80% of deaths occurring in low- and medium- income countries, which amounts to 66.3% of the total disease burden. The main NCDs are cardiovascular diseases, cancer, respiratory chronic diseases and diabetes[2].

In recent years, the simultaneous occurrence of two or more chronic diseases, referred to as multimorbidity, has been the focus of studies around the world as this condition severely affects individuals [3]. The multimorbidity pattern can be influenced by several factors, of which some are modifiable and preventable if strategies towards the improvement of health habits are promoted to the population, such as campaigns against tobacco consumption and sedentary habits, and promotion of the benefits of a healthy diet [4].Studies report that socioeconomic, cultural, geographic and lifestyle-related factors can affect the occurrence and intensity of multimorbidity [5–7].

In Brazil, in 2013, the prevalence of self-reported multimorbidity was 23.6%in individuals older than 18, and higher among women (28.4%) when compared with men (18.2%) [8]. In clinical practice, multimorbidity has been under-diagnosed and underestimated. It is necessary to change the paradigm of interventions focused on a single illness only, widening the approach to encompass multiple morbidities, reducing the chances of polypharmacy (prescription of multiple medications) and repetitive referrals to specialized care, which could increase the risk of adverse pharmaceutical effects[9].

The aim of this study was to define the main grouping patterns of chronic diseases and the relationship with socioeconomic and lifestyle-related factors in the Brazilian population.

## Methods

This cross-sectional study was based on data from the National Health Survey of 2013, a nation-wide survey carried out in the Brazilian population by the Ministry of Health and the Brazilian Institute of Geography and Statistics (IBGE). The survey was part of the integrated system of home censuses, and generated data on the health situation, lifestyle, and use of health services of the population. The complex sample design is detailed elsewhere [10].This cross-sectional study was based on data from questionnaires answered by 60,202 individuals over the age of 18. Multimorbidity was evaluated by the presence of two or more self-reported chronic diseases (within a group of 14 chronic conditions). The interviewees were asked whether they had a previous diagnosis of chronic diseases such as hypertension, diabetes, hypercholesterolemia, asthma, cardiac diseases, mental diseases, cerebrovascular accident (CVA), arthritis, chronic obstructive pulmonary disease (COPD), spinal issues, depression, cancer, renal insufficiency and work-related musculoskeletal disorders (WRMD). The independent variables related to lifestyle were the consumption of tobacco; consumption of alcohol, utilizing the World Cancer Research Fund parameter for moderate consumption limited to one serving of alcohol a day for women and two servings for men—servings that exceeded this number were considered as excessive consumption [11]; healthy dietary habits, considering the World Health Organization (WHO) standards: daily consumption of 400 g or five servings of healthy foods a day (vegetables, natural juices, salads and fruit)[12].Practice of physical activities was categorized considering total minutes dedicated to physical activity, with combined categories of minutes per week dedicated to physical exercise or sports, leisure, physical efforts at work, work commuting or other displacements, and home chores that involved physical effort, utilizing the WHO 2010 standard of at least 150 minutes of moderate physical activity per week or 75 minutes of vigorous activity per week for adults, in sessions at least 10 minutes long [13]. The Body Mass Index (BMI) was calculated from self-reported height and weight, according to the WHO classification [13].

Self-reported height and weight presented 32% of missing information, which was managed using Multiple Imputation by Chained Equations (MICE). Variables related to missing data, along with height and weight values, were utilized to build the MICE models. Thirty-two datasets were imputed, corresponding to 32% of missing data [14], to improve reliability.

Prevalence of each study variable was calculated as percentages with 95% CI, considering the complex sample design.

Different multimorbity patterns were defined through cluster analysis of the hierarchical type, which is ideal to define the similarity between diseases without any influence of previous hypotheses. The variables included were the 14 chronic conditions aforementioned. Cluster analysis enables consideration beyond the pairs of comorbidities, and analyzes how diseases tend to occur in conjunction with each other. Application of this technique facilitates further understanding on how the association between several diseases occurs in a given population, and how the distribution of the diseases found in each cluster should be significantly different from the random distribution. A correlation matrix was computed for all the conditions using the Yule's Q measure of association, with average linkage as a combination method. Yule's Q is a similarity measurement of association that calculates the strength of association between binary variables. The dendogram and the theoretical model of approximation among chronic diseases based on other studies were used to define the number of clusters[15].

The prevalence of each cluster was explored using modified Poisson regression models with robust error variance to obtain prevalence ratios. Multivariate models were built each one of the clusters using a forward stepwise approach, maintaining independent variables in the model with p-value <0.05 in Wald tests, pertaining to the specific clusters was the outcome. The Prevalence Ratio (PR) was obtained from the event classification (exposed and non-exposed). Analyses were carried out withStata14 (Stata Corp. Inc. TX, USA, version 14), utilizing the survey module for complex samples and considering multiple imputation.

Data supporting the conclusions of this study are available in the public domain (IBGE website:http://www.ibge.gov.br/home/estatistica/populacao/pns/2013/).

## Results

Univariate analysis provided baseline characteristics for the study, describing socioeconomic, demographic and lifestyle-related characteristics (Table 1).

**Table 1:** Sample size and distribution (% - 95% CI) of study population, according to socioeconomic, demographic and lifestyle-related characteristics.

Characteristics		n	% (95% CI^a^)
Sex	Male	25.920	43.0(42.6–43.4)
	Female	34.282	56.9(56.5–57.3)
Age	18–29	14.321	23.8(23.4–24.1)
	30–39	14.269	23.7(23.3–24.0)
	40–49	11.405	18.9(18.6–19.2)
	50–59	9.030	14.9(14.7–15.2)
	60 or over	11.177	18.5(18.2–18.8)
Education level (years of study)	0 to 3	5.994	9.9(9.7–10.1)
	4 to 7	22.384	37.1(36.7–37.5)
	8 to 10	20.026	33.2(32.8–33.6)
	11 or more	11.798	19.5(19.2–19.9)
Skin color^b^	White	24.106	40.0(39.6–40.4)
	Black	5.631	9.3(9.1–9.5)
	Yellow	533	0.8(0.8–0.9)
	*Parda*^*c*^	29.512	49.0(48.6–49.4)
	Indigenous	417	0.6(0.6–0.7)
Residence area	Urban	49.425	81.7(81.4–82.1)
	Rural	10.957	18.2(17.8–18.5)
Private health insurance	Yes	16.368	27.2(26.8–27.5)
	No	43.834	72.8(72.4–73.1)
Living with partner/spouse	Yes	34.522	57.3(56.9–57.7)
	No	25.680	42.6(42.2–43.0)
Work situation	Working	36.422	60.5(60.1–60.9)
	Not working	23.760	39.4(39.0–39.8)
Smoking	Never smoked	41.215	68.4(68.0–68.8)
	Ex-smoker	10.258	17.0(16.7–17.3)
	Current smoker	8.729	14.4(14.2–14.7)
Alcohol intake	Absteiner	46.976	78.0(77.6–78.3)
	Moderate	9.017	14.9(14.6–15.2)
	Excessive	4.209	6.9(6.7–7.1)
Practice of physical activity	Sufficient	31.613	52.5(52.1–52.9)
	Insufficient	10.800	17.9(17.6–18.2)
	None	17.789	29.5(29.1–29.9)
Fruits and vegetables intake	Sufficient	17.886	29.7(29.3–30.0)
	Insufficient	41.158	68.3(67.9–68.7)
	None	1.158	1.9(1.8–2.0)
BMI^d^	Underweight	1.531	4.6(4.5–4.6)
	Normal weight	26.446	43.2(43.1–43.4)
	Overweight	21.271	34.5(34.4–34.6)
	Obesity	10.954	17.5(17.4–17.6)

a CI: confidence interval.

b Unknown category was ommited (n = 3; % (95% CI) = 5E-05(-6.56E-06, 1E-04).

c Brazilians of mixed ethnic ancestries.

d BMI: Body mass index, derived from imputed dataset.

**Fig 1:**
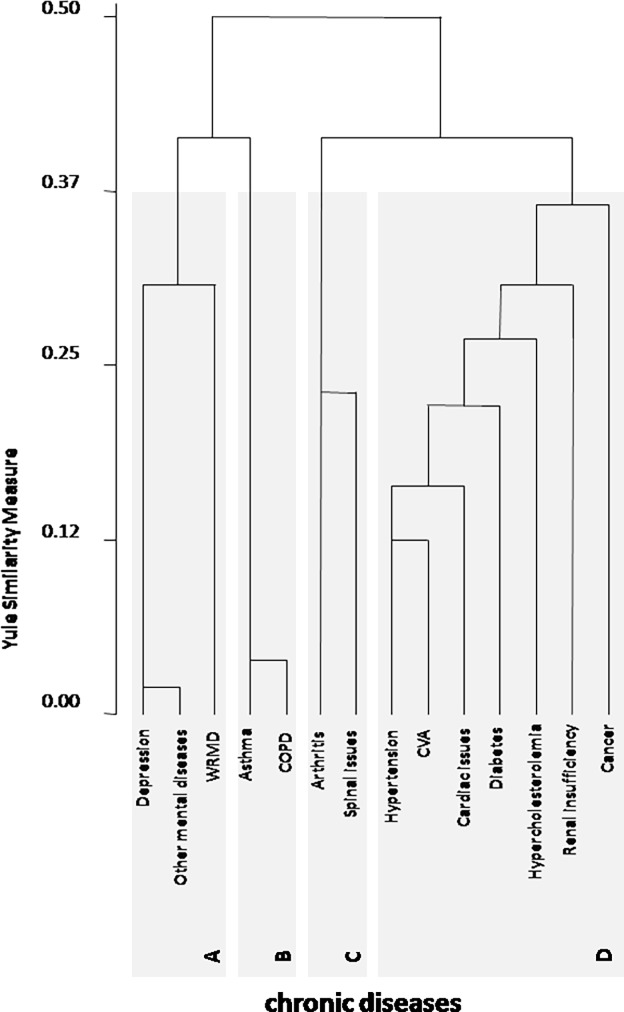
Clusters of disease in Brazil. Cerebrovascular accident (CVA), chronic obstructive pulmonary disease (COPD), work-related musculoskeletal disorders (WRMD). A: Mental/occupational Cluster. B: Respiratory Cluster. C: Musculoskeletal Cluster. D: Cardiometabolic/cancer Cluster.

**Fig 2:**
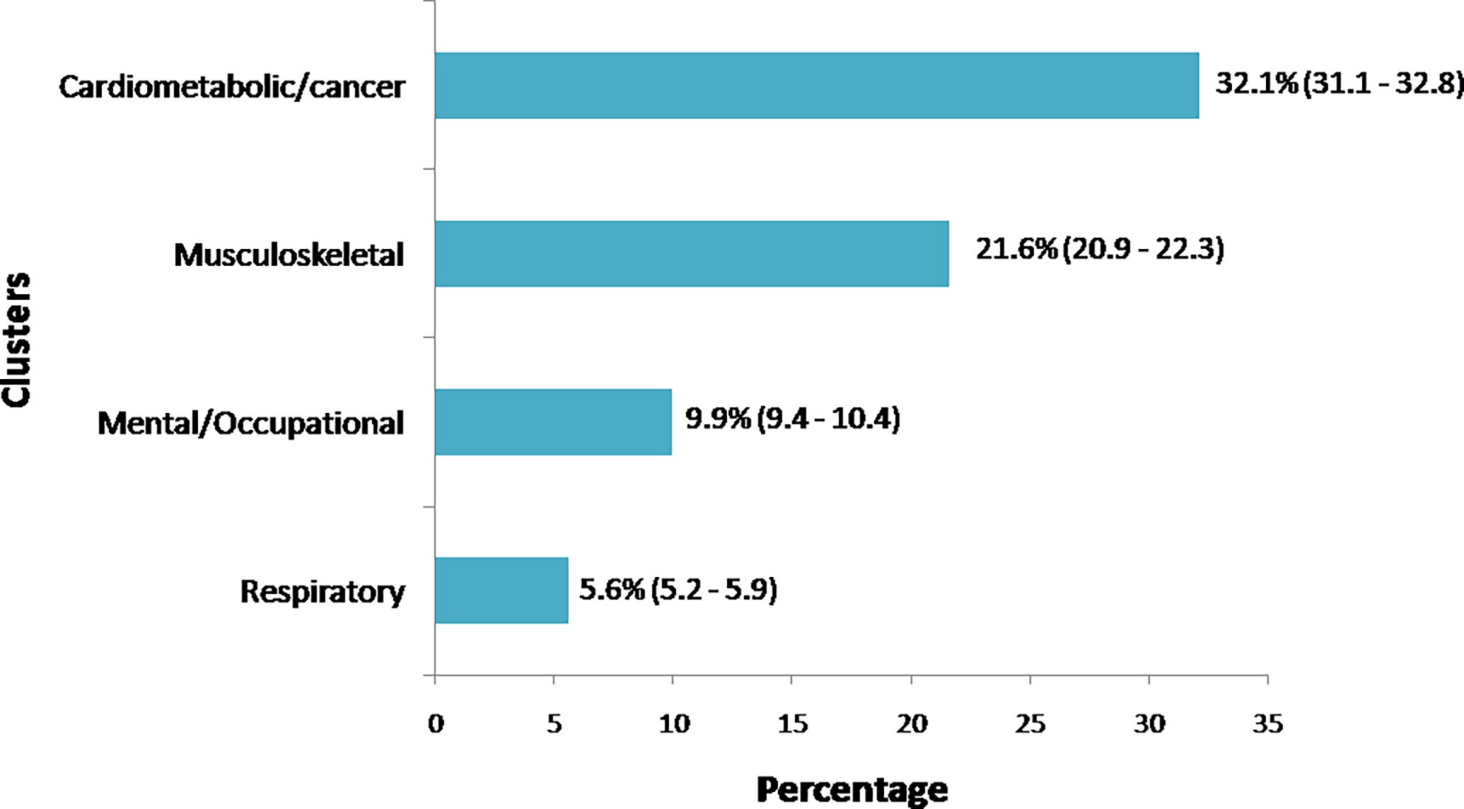
Prevalence (confidence interval 95%) of chronic diseases, Brazil.

Cluster analysis identified four clusters of disease (Fig 1). The first cluster was denominated cardiometabolic/cancer (prevalence of 32.1% in the general population–Fig 2) and was constituted by seven diseases: the most prevalent diseases, which included hypertension, CVA, cardiac issues, hypercholesterolemia and diabetes, besides renal insufficiency and cancer. The second cluster was denominated mental/occupational (9.9% of the general population) and was constituted by WRMD, depression, and other mental diseases. The third cluster was constituted by two diseases: spinal issues and arthritis, and was denominated musculoskeletal (prevalence of 21.6%). The fourth cluster included two diseases: asthma and chronic obstructive pulmonary disease, and was denominated respiratory (prevalence of 5.6%).

In the cardiometabolic/cancer cluster (hypertension, CVA, cardiac issues, hypercholesterolemia, diabetes, renal insufficiency and cancer) a 32.1% prevalence was observed, which increased with age and was higher in women. Individuals over the age of 60, without private health insurance, living with spouses or a partner, living in urban locations, the not working and those with low education levels presented higher prevalence in this cluster. Higher prevalence was observed in ex-smokers, in those who did not consume alcoholic beverages or exercised, and in the obese. Prevalence was lower among individuals who never smoked, in those who practiced physical activities, and in those who did not consume fruit, legumes and vegetables. Regarding multivariate analysis, a higher prevalence of the cardiometabolic/cancer cluster was associated with the female sex, age group over 60, low education levels, private health plans, not working, past consumption of tobacco and obesity (Table 2).

**Table 2:** Prevalence (%) and association between socioeconomic and lifestyle-related variables and the cardiometabolic/cancer cluster, crude and multivariate prevalence ratios.

		Cardiometabolic/Cancer cluster				
		Prevalence %	Crude PR^a^	*p-value*	Multivariate Adjusted PR^a^	*p-value*
		(95% CI^b^)	(95% CI^b^)		(95% CI^b^)	
Sex	Male	27.8(26.8–28.7)	1.00	<0.001	1.00	<0.001
	Female	35.9(35.0–36.8)	1.29 (1.24–1.34)		1.22 (1.17–1.27)	
Age group	18 to 24	5.7(4.9–6.6)	1.00	<0.001	1.00	<0.001
	25 to 39	15.0(14.2–15.9)	2.62 (2.22–3.09)		2.33 (1.97–2.75)	
	40 to 59	41.9(40.7–43.9)	7.29 (6.24–8.52)		5.79 (4.95–6.78)	
	60 or more	66.6(65.0–68.2)	11.60 (9.93–13.53)		8.56 (7.32–10.02)	
Education level (years of study)	11 or more	26.7(25.3–28.2)	1.00	<0.001	1.00	<0.001
	8 to 10	22.3(21.3–23.3)	0.83(0.78–0.89)		1.04 (0.97–1.13)	
	4 to 7	39.9(38.7–41.1)	1.49(1.40–1.58)		1.07 (1.01–1.14)	
	0 to 3	48.6(46.3–50.9)	1.81(1.69–1.95)		0.95 (0.89–1.01)	
Skin color	White	33.6 (32.6–34.7)	1.00	<0.001	_	
	Other^c^	30.7 (29.2–31.6)	0.91 (0.87–0.94)		_	
Residence area	Rural	28.6 (27.2–30.2)	1.00	<0.001	1.00	<0.001
	Urban	32.6 (31.9–33.4)	1.13 (1.07–1.20)		1.12 (1.06–1.18)	
Living with partner/spouse	Yes	34.7 (33.8–35.6)	1.00	<0.001	1.00	0.003
	No	27.9 (26.9–29.2)	0.80 (0.77–0.84)		0.94 (0.90–0.98)	
Private health insurance	Yes	30.5(29.7–31.4)	1.00	<0.001	1.00	<0.001
	No	35.5(34.3–36.8)	1.16(1.11–1.21)		1.10 (1.05–1.15)	
Work situation	Working	25.3(24.5–26.2)	1.00	<0.001	1.00	<0.001
	Not working	42.8(41.7–44.0)	1.68(1.62–1.75)		1.13 (1.08–1.18)	
Smoking	Never	28.1(27.3–28.9)	1.00	<0.001	1.00	<0.001
	Ex-smoker	48.4(46.6–50.1)	1.71(1.64–1.79)		1.23 (1.18–1.28)	
	Current	30.8(29.0–32.7)	1.09(1.02–1.16)		1.04 (0.98–1.11)	
Alcohol intake	Abstainer	34.1(33.2–34.9)	1.00	<0.001	_	
	Moderate	25.8(24.3–27.3)	0.75(0.71–0.80)		_	
	Excessive	25.5(23.3–27.8)	0.74(0.68–0.82)		_	
Practice of physical activity	Sufficient	28.7(27.8–29.5)	1.00	<0.001	_	
	Insufficient	33.0(31.4–34.7)	1.15(1.08–1.21)		_	
	None	38.0(36.7–39.3)	1.32(1.26–1.38)		_	
Fruit and vegetables intake	Sufficient	32.6(31.3–33.9)	1.00	<0.001	1.00	0.017
	Insufficient	32.1(31.2–32.9)	0.98(0.93–1.03)		0.77 (0.92–1.00)	
	None	21.5(18.0–25.6)	0.66(0.55–0.79)		0.79 (0.67–0.94)	
BMI^d^	Normal	32.1(31.4–32.9)	1.00	<0.001	1,00	<0.001
	Underweight	1.7 (1.5–2.0)	0.94 (0.77–1.16)		0.95 (0.79–1.14)	
	Overweight	38.3(37.5–39.1)	1.58 (1.49–1.67)		1.34 (1.28–1.41)	
	Obesity	27.7(27.0–28.4)	2.22 (2.10–2.34)		1.73 (1.64–1.82)	
Total		32.1(31.3–32.8)				

^a^ PR: Prevalence Ratio

^b^ CI: Confidence Interval

^c^ Other: Black, Yellow, *Parda*,Indigenous

^d^ BMI: Body mass index, derived from imputed dataset.

In the mental/occupational cluster (WRMD, depression and mental diseases), a 9.9% prevalence was verified, which was higher in the economically-active age group (40–59 years of age). Women, individuals without private health insurance, those living in urban areas and the not working presented higher prevalence. Higher prevalence was also observed among alcohol abstainers and in the obese, and lower prevalence was observed in those who had never smoked tobacco. After multivariate analysis, higher prevalence of the mental/occupational cluster was associated with the female sex, the age group 40–49 years old, white skin color, those who did not live with spouses or partners, private health insurance, not working, smokers and the obese (Table 3).

**Table 3:** Prevalence (%) and association between socioeconomic and lifestyle-related variables and the mental/occupational cluster, crude and multivariate prevalence ratios.

		Mental/Occupational cluster				
		Prevalence %	Crude PR^a^	*p-value*	Multivariate Adjusted PR^a^	*p-value*
		(95% CI^b^)	(95% CI^b^)		(95% CI^b^)	
Sex	Male	5.6(5.1–6.2)	1.00	<0.001	1.00	<0.001
	Female	13.7(12.9–14.5)	2.42(2.18–2.69)		2.38 (2.12–2.66)	
Age group	18 to 24	4.6(3.8–5.7)	1.00	<0.001	1.00	<0.001
	25 to 39	8.9(8.2–9.7)	1.91 (1.53–2.37)		1.84 (1.47–2.29)	
	40 to 59	12.8(11.8–13.7)	2.74 (2.22–3.37)		2.31 (1.86–2.87)	
	60 or more	10.9(9.8–12.1)	2.34 (1.88–2.92)		1.78 (1.41–2.24)	
Education level (years of study)	11 or more	11.1(10.0–12.2)	1.00	<0.001	1.00	0.003
	8 to 10	8.5(7.8–9.3)	0.77(0.67–0.87)		0.77 (0.63–0.95)	
	4 to 7	10.9(10.1–11.7)	0.98(0.87–1.10)		1.01 (0.89–1.15)	
	0 to 3	8.4(7.2–9.7)	0.75(0.63–0.90)		0.89 (0.78–1.01)	
Skin color	White	11.6 (10.8–12.4)	1.00	<0.001	1.00	<0.001
	Other^c^	8.4 (7.8–9.0)	0.71 (0.65–0.78)		0.77 (0.70–0.85)	
Residence area	Rural	6.8 (5.9–7.8)	1.00	<0.001	1.00	<0.001
	Urban	10.4 (9.8–11.0)	1.52 (1.30–1.78)		1.32 (1.12–1.55)	
Living with partner/spouse	Yes	9.8 (9.2–10.5)	1.00	0,695	1.00	0,013
	No	10.0 (9.3–10.8)	1.02 (0.93–1.10)		1.12 (1.02–1.23)	
Private health insurance	Yes	8.9(8.4–9.5)	1.00	<0.001	1.00	<0.001
	No	12.0(11.1–13.1)	1.34(1.22–1.48)		1.22 (1.09–1.35)	
Work situation	Working	8.6(8.1–9.2)	1.00	<0.001	1.00	<0.001
	Not working	11.9(11.0–12.7)	1.37(1.26–1.49)		1.19 (1.07–1.31)	
Smoking	Never	8.9(8.3–9.5)	1.00	<0.001	1.00	<0.001
	Ex-smoker	12.1(11.0–13.3)	1.35(1.21–1.50)		1.39 (1.24–1.55)	
	Current	11.8(10.6–13.1)	1.32(1.18–1.48)		1.50 (1.34–1.68)	
Alcohol intake	Abstainer	10.5(9.9–11.1)	1.00	<0.001	_	
	Moderate	8.1(7.2–9.2)	0.77(0.68–0.88)		_	
	Excessive	7.3(6.0–9.0)	0.69(0.56–0.86)		_	
Practice of physical activity	Sufficient	9.8(9.2–10.5)	1.00	0.462	_	
	Insufficient	10.5(9.4–11.7)	0.97 (0.88–1.08)		_	
	None	9.6(8.7–10.6)	1.06 (0.94–1.20)		_	
Fruit and vegetables intake	Sufficient	10.0 (9.2–10.9)	1.00	0.504	_	
	Insufficient	9.9 (9.2–10.5)	0.98 (0.88–1.08)		_	
	None	8.4 (6.2–11.2)	0.83 (0.61–1.13)		_	
BMI^d^	Normal	38.9 (37.5–40.3)	1.00	<0.001	1.00	<0.001
	Underweight	2.6 (2.4–2.7)	0.86 (0.58–1.27)		0.86 (0.59–1.27)	
	Overweight	35.5 (34.1–37.0)	1.13 (1.01–1.25)		1.09 (0.98–1.21)	
	Obesity	23.4 (22.2–24.7)	1.53 (1.36–1.72)		1.34 (1.19–1.50)	
Total		9.9(9.4–10.4)				
			

^a^ PR: Prevalence Ratio

^b^ CI: Confidence Interval

^c^ Other: Black, Yellow, *Parda*, Indigenous

^d^ BMI: Body mass index, derived from imputed dataset.

In the musculoskeletal cluster (spinal issues and arthritis), a 21.6% prevalence was observed, which increased with age for men and women, but affected women more pronouncedly. People over the age of 60, living with a partner or spouse, residing in rural locations, not working and those with low education levels presented higher prevalence in this cluster. Multivariate analysis revealed associations between the musculoskeletal cluster and the female sex, age group over 60 years old, low education levels, those living with spouses or partners, consumption of tobacco, and obesity (Table 4).

**Table 4:** Prevalence (%) and association between socioeconomic and lifestyle-related variables and the musculokeletal cluster, crude and multivariate prevalence.

						
		Musculokeletal cluster				
		Prevalence %	Crude PR^a^	*p-value*	Multivariate Adjusted PR^a^	*p-value*
		(95% CI^b^)		* *	(95% CI^b^)	* *
Sex	Male	17.5(16.6–18.4)	1.00	<0.001	1.00	<0.001
	Female	25.2(24.3–26.2)	1.44(1.36–1.52)		1.50 (1.42–1.58)	
Age group	18 to 24	8.5(7.3–9.8)	1.00	<0.001	1.00	<0.001
	25 to 39	14.3(13.4–15.2)	1.68 (1.45–1.94)		1.49 (1.28–1.73)	
	40 to 59	27.0(25.9–28.2)	3.18 (2.75–3.67)		2.49 (2.14–2.90)	
	60 or more	35.8(34.2–37.4)	4.20 (3.64–4.85)		3.26 (2.80–3.79)	
Education level (years of study)	11 or more	15.9(14.7–17.2)	1.00	<0.001	1.00	<0.001
	8 to 10	16.4(15.5–17.5)	1.03(0.93–1.14)		1.43 (1.28–1.59)	
	4 to 7	26.7(25.7–27.9)	1.68(1.54–1.82)		1.34 (1.24–1.46)	
	0 to 3	32.4(30.2–34.7)	2.03(1.83–2.25)		1.12 (1.02–1.23)	
Skin color	White	22.6 (21.6–23.6)	1.00	0.005	1.00	0.014
	Other^c^	20.8 (19.9–21.7)	0.92 (0.87–0.97)		0.93 (0.87–0.98)	
Residence area	Rural	24.4 (22.7–26.3)	1.00	<0.001	_	
	Urban	21.2 (20.4–21.9)	0.86 (0.79–0.94)		_	
Living with partner/spouse	Yes	23.6 (22.7–24.5)	1.00	<0.001	1.00	<0.001
	No	18.5 (17.7–19.4)	0.78 (0.74–0.82)		0.86 (0.81–0.91)	
Private health insurance	Yes	21.6 (20.8–22.5)	1.00	0.874	_	
	No	21.5 (20.3–22.8)	0.99 (0.93–1.06)		_	
Work situation	Working	26.4 (25.4–27.4)	1.00	<0.001	_	
	Not working	18.6 (17.8–19.5)	1.41 (1.34–1.49)		_	
Smoking	Never	18.6 (17.8–19.4)	1.00	<0.001	1.00	<0.001
	Ex-smoker	30.9 (29.3–32.5)	1.66(1.56–1.76)		1.33(1.25–1.42)	
	Current	24.6 (23.0–26.3)	1.32(1.22–1.42)		1.25 (1.16–1.35)	
Alcohol intake	Abstainer	22.6 (21.8–23.4)	1.00	<0.001	_	
	Moderate	19.2 (17.7–20.8)	0.85(0.78–0.92)		_	
	Excessive	17.0 (15.1–19.1)	0.75(0.66–0.84)		_	
Practice of physical activity	Sufficient	21.6 (27.8–29.5)	1.00	0.782	1.00	<0.001
	Insufficient	21.3 (19.8–22.8)	0.97 (0.90–1.05)		0.89 (0.83–0.96)	
	None	21.5 (20.9–22.7)	0.98 (0.92–1.04)		0.80 (0.76–0.85)	
Fruit and vegetables intake	Sufficient	21.1 (19.9–22.2)	1.00	0.232	_	
	Insufficient	21.9 (21.1–22.8)	1.03 (0.97–1.10)		_	
	None	19.0 (15.4–23.2)	0.90 (0.73–1.11)		_	
BMI^d^	Normal	38.8 (37.9–39.8)	1.00	<0.001	1.00	<0.001
	Underweight	2.1 (1.8–2.4)	0.90 (0.71–1.14)		0.88 (0.70–1.12)	
	Overweight	36.1 (35.1–37.0)	1.18 (1.10–1.26)		1.07 (1.01–1.15)	
	Obesity	22.8 (22.0–23.6)	1.43 (1.33–1.53)		1.20 (1.12–1.29)	
Total		21.6(20.9–22.3)				

^a^ PR: Prevalence Ratio.

^b^ CI: Confidence Interval

^c^ Other: Black, Yellow, *Parda*, Indigenous

^d^ BMI: Body mass index, derived from imputed dataset.

Regarding the respiratory cluster (asthma and COPD), a 5.6% prevalence was observed. There was higher prevalence among women, in people over the age of 60, among not working and in those residing in urban locations. Higher prevalence was observed in individuals without private health insurance, ex-smokers and in those with normal BMI, and lower prevalence was identified in those who never smoked and the underweight. After adjustment, there was an association between the respiratory cluster and the female sex, the age group 18–29 years old, consumption of tobacco and obesity (Table 5).

**Table 5:** Prevalence (%) and association between socioeconomic and lifestyle-related variables and the respiratory cluster, crude and multivariate prevalence ratios.

						
		Respiratory cluster				
		Prevalence %	Crude PR^a^	*p-value*	Multivariate Adjusted PR^a^	*p-value*
		(95% CI^b^)		* *	(95% CI^b^)	* *
Sex	Male	4.8 (4.4–5.3)	1.00	<0.001	1.00	<0.001
	Female	6.3 (5.8–6.8)	1.31 (1.16–1.47)		1.39 (1.22–1.57)	
Age group	18 to 24	6.2 (5.3–7.2)	1.00	<0.001	1.00	<0.001
	25 to 39	4.9 (4.4–5.5)	0.80 (0.66–0.96)		0.74 (0.61–0.89)	
	40 to 59	4.9 (4.4–5.4)	0.79 (0.66–0.94)		0.66 (0.55–0.78)	
	60 or more	7.5 (6.7–8.5)	1.22 (1.01–1.47)		1.03 (0.85–1.26)	
Education level (years of study)	11 or more	6.4 (5.6–7.3)	1.00	0.052	_	
	8 to 10	5.1(4.6–5.7)	0.80 (0.62–1.03)		_	
	4 to 7	5.8 (5.2–6.4)	0.90 0.76–1.07)		_	
	0 to 3	5.1 (4.2–6.3)	0.80 (0.68–0.94)		_	
Skin color	White	6.3 (5.8–6.8)	1.00	<0.001	1.00	<0.001
	Other^c^	5.0 (4.6–5.4)	0.79 (0.70–0.88)		0.80 (0.71–0.90)	
Residence area	Rural	3.9 (3.3–4.5)	1.00	<0.001	1.00	<0.001
	Urban	5.9 (5.5–6.3)	1.51 (1.27–1.80)		1.45 (1.21–1.72)	
Living with partner/spouse	Yes	5.4 (4.9–5.8)	1.00	0.132	_	
	No	5.9 (5.4–6.5)	1.09 (0.97–1.23)		_	
Private health insurance	Yes	5.3(4.9–5.7)	1.00	0.005	_	
	No	6.3(5.7–7.0)	1.19(1.05–1.35)		_	
Work situation	Working	5.1 (4.7–5.6)	1.00	0.001	_	
	Not working	6.3 (5.7–6.9)	1.22 (1.08–1.38)		_	
Smoking	Never	4.8(4.5–5.2)	1.00	0.001	1.00	<0.001
	Ex-smoker	7.3(6.5–8.3)	1.50(1.30–1.73)		1.56 (1.34–1.82)	
	Current	6.9(6.0–7.9)	1.41(1.21–1.66)		1.62 (1.38–1.91)	
Alcohol intake	Abstainer	5.7 (5.4–6.2)	1.00	0.242	_	
	Moderate	5.0 (4.3–5.8)	0.87 (0.74–1.03)		_	
	Excessive	5.3 (4.2–6.6)	0.92 (0.72–1.16)		_	
Practice of physical activity	Sufficient	5.9 (5.4–6.3)	1.00	0.164	1.00	0.013
	Insufficient	5.4 (4.7–6.1)	0.91 (0.79–1.05)		0.89 (0.77–1.02)	
	None	5.2 (4.6–5.9)	0.88 (0.76–1.01)		0.81 (0.70–0.93)	
Fruit and vegetables intake	Sufficient	5.4 (4.8–6.0)	1.00	0.729	_	
	Insufficient	5.7 (5.2–6.1)	1.05 (0.91–1.21)		_	
	None	5.8 (3.6–9.2)	1.08 (0.67–1.75)		_	
BMI^d^	Normal	40.2 (38.4–42.0)	1.00	<0.001	1.00	<0.001
	Underweight	2.9 (2.2–3.6)	1.39 (0.93–2.09)		1.37 (0.91–2.06)	
	Overweight	33.9 (32.1–35.8)	1.06 (0.92–1.22)		1.10 (0.95–1.28)	
	Obesity	22.8 (21.2–24.3)	1.39 (1.19–1.63)		1.43 (1.22–1.67)	
Total		5.6 (5.2–5.9)				
			

^a^ PR: Prevalence Ratio.

^b^ CI: Confidence Interval

^c^ Other: Black, Yellow, *Parda*, Indigenous

^d^ BMI: Body mass index, derived from imputed dataset.

## Discussion

Four disease clusters were identified in the Brazilian population and the most prevalent were, respectively: cardiometabolic diseases/cancer, musculoskeletal diseases, mental/occupational diseases and respiratory diseases. Lower prevalence diseases, such as renal insufficiency and cancer, were included in the cardiometabolic cluster along with the most frequent diseases: cardiovascular diseases and diabetes.

Similar results of disease clusters were obtained in other studies carried out in developed countries [16–19]. Despite the methodological variability among studies, it was possible to observe similarities between the present study and the patterns described in scientific literature. A systematic review demonstrated relevant similarities for three groups of patterns: the first comprehended a combination of cardiovascular and metabolic diseases; the second was related to mental health issues; and the third was related to musculoskeletal disorders [20]. Another study described multimorbidity patterns in adults over 50 years of age in low, intermediate, and high-income countries, analyzing data from the collaborative research project on aging in Europe (Finland, Poland, and Spain) and from the WHO on global and adult aging processes (China, Ghana, India, Mexico, Russia and South Africa). The main patterns identified were: cardio-respiratory (cardiac issues, asthma and COPD), metabolic (diabetes, obesity and hypertension), and mental-articular (arthritis and depression)[21]. Rezewuska et al. (2017) conducted a study in the Brazilian population to identify multimorbidity patterns, but focused on identifying factors related to any mental health disorder, which limits the comparison with the findings presented herein[22].

This study focused on the clustering of diseases and the factors associated with each disease. These factors were classified according to healthcare service planning: non-modifiable factors (age, sex and skin color), contextual and historical factors that are influenced by broader social policies and expressed as social inequities (health insurance, area of residence, education, work situation) and lifestyle factors (smoking, alcohol consumption, physical activity practice, consumption of fruits and vegetables, and obesity).

Actions directed to non-modifiable factors should be based on the principles of access to health services, which are universality and equity. A higher prevalence of all clusters was observed in women, when compared to men. The differences observed are possibly related to the more intense utilization of health services by women, which increases the opportunities of diagnosis and increases familiarity with medical terms and the signs and symptoms associated with diseases [23]. In Brazil, public policies are mainly directed to the female health, including programs for cervical and breast cancer prevention, pre-natal humanization programs, among others. Policies directed to male health are still being consolidated and require better development.

The high prevalence of disease clusters in the elderly population requires special attention in the field of public health. In recent years, especially after the 1980's, the effect of decreasing fecundity levels and mortality in Brazil resulted in the transformation of a young population into an aging population [24]. In 2006, the National Health Policy for the Elderly was created in Brazil, with special mention to the Plan of Reorganization of Attention to Arterial Hypertension and Diabetes, which enables monitoring and treatment of these conditions in a regular and organized basis [25]. However, the multimorbidity aspect is contemplated in a very superficial way within this policy (comorbidity only), without referral to specific protocols for the elderly.

A significant contingent of the economically-active Brazilian population, within the age group of 40–59 years old, was observed in the mental/occupational cluster. Regarding the role of the State in preventing work-related diseases and its integral attention, there are flaws and contradictions in public regulations, with no articulated inter-sector actions in the country, with few preventive actions[26]. Despite the initiative of the Ministry of Health to institute the Work-Related Health National Policy, surveillance of the health of employees/workers is still incipient. Employee health care should be essentially under the competence of work-related health centers, but these depend on other services that do not focus on work-related risk prevention [27].

The Brazilian Unified Health System (Brazil's publicly funded health care system, SUS) was created in 1988. SUS provides universal and free access to the entire population (more than 208 million people). Private health insurances provide healthcare to 25% of the population, which are those with better economic conditions. Individuals that can afford private health insurance presented lower prevalence of all clusters, in comparison with the users of the public healthcare system[28]. Private health insurances represent better socioeconomic conditions, and probably better health. This highlights the challenge of accessing primary care in the public health system. In addition, the concentration of diagnosis and treatment services, whether public or private, is higher in urban areas. Probably for this reason, living in urban areas was associated with different clusters, with an exception for the musculoskeletal cluster, which is constituted by diseases that are easily diagnosed.

The education level was associated with multimorbidity clusters, with higher prevalence among people with less years of study, especially for the cardiometabolic and musculoskeletal clusters. Education is a social determinant directly related to income inequalities, with strong impact on mortality and morbidity in different population groups [29]. Some authors claim that the association between education and multimorbidity is not well described, however the study of the Heidelberg cohort of the European Prospective Research on Cancer and Nutrition (EPIC) reports that intermediate factors can explain such associations. In their findings, compared to the highest educational category, the lowest education levels were statistically associated with a greater chance of multimorbidity in males, attenuated after adjusting the model for other factors such as BMI and smoking[30].

Not working individuals presented higher prevalence in the cardiometabolic/cancer and mental/occupational clusters, when compared with employed individuals. Multimorbidity is associated with an increase in functional decline rates [17], and therefore this higher prevalence could be attributed to the fact that people with these multimorbidity patterns could have compromised working capacities. However, the cross-sectional design of this study is not sufficient to confirm whether chronic diseases occurred before or after unemployment.

Multimorbidity patterns are influenced by the lifestyle factors of individuals at younger ages, which could include risk factors for specific conditions and cause chronic problems [1]. Common sub-adjacent etiopathogenic factors could play an important role in the formation of clusters [24]. When analyzing the factors related to lifestyle in clusters, it was possible to observe a higher prevalence of clusters in less-healthy lifestyles. In Spain, a study based on the 2009 European Health Survey on individuals 15 years of age and older verified the existence of an inverse association between multimorbidity and the practice of physical activities, also observing that the presence of functional limitations was related to lower levels of physical exercise [31].The results presented herein showed a higher prevalence of physical activity in the musculoskeletal and respiratory disease clusters, which could be explained by reverse causality, a limitation of sectional studies.

Some risk factors were common to all clusters, such as smoking and obesity. Regarding smoking, a global effort is observed, involving governmental and non-governmental organisms, health professionals and the civil society, to regulate and control tobacco, to discourage its consumption, preventing initiation and stimulating quitting [32]. Despite the implementation of health policies against smoking, this is still a preponderant risk factor for chronic diseases [33], with a 14% prevalence in populations aged 18 years and over, in 2013. In recent years, Brazil has approved several regulations on smoking tobacco in public spaces, on advertising, with tax raises and regulations on the use of additives, even enforcing policies to substitute the agriculture of tobacco for other crops. However, recent data show deficiencies in the policies against smoking, especially in the counseling of health professionals on quitting smoking [32].

In the epidemiological scenario of NCDs, obesity is highlighted because it is simultaneously a disease and a risk factor for other diseases within the group, such as hypertension and diabetes. In Brazil, approximately 15% of adults are obese, and approximately half the population over 20 years old is overweight [34]. Similar results have been reported in other countries. A study evaluating the association between body mass index and prevalence of multimorbity in low- and middle-income countries observed that the prevalence of multimorbidity is 1.5 times higher in the obese than in normal weight individuals. Moreover, obesity was independently associated with multimorbidity in six low- and middle-income countries, with a prevalence of 37% in the obese population[35]. Other authors also describe obesity as one of the main predictors of multimorbidity[36]. The clear difficulty in combating obesity is one of the main challenges in the fight against NCDs, and consequently, against multimorbidity, given the complexity of weight gain, influenced by globalization regarding dietary patterns and contemporary lifestyles [37–38].

The main strength of this study is the complex survey design, which makes it representative of the Brazilian population. This study presented limitations due to the utilization of self-reported diagnosis data, with the possibility of under-registry that could underestimate the real prevalence of multimorbidity clusters. In addition, some social groups could find more obstacles to accessing healthcare inequalities. The cross-sectional design of the study hinders any causal inference.

The results presented herein show that different groups of diseases (cardiometabolic diseases / cancer, musculoskeletal diseases, mental / occupational diseases and respiratory diseases) identified in the Brazilian population were associated with being a current or former smoker and high BMI. Contextual and historical factors related to social policies, such as education and employment, require further longitudinal studies. These factors must be considered when planning actions and elaborating protocols to manage multimorbidity. Challenging aspects include the involvement of those acting in the process, the incorporation of the knowledge generated and its applications in daily professional practices, and intersectoral actions that target the reduction of social inequities.

## Supporting information

S1 File Collinearity tests. (TXT)
